# Rising incidence of radiation pneumonitis after adjuvant durvalumab in NSCLC patients treated with concurrent chemoradiotherapy

**DOI:** 10.2340/1651-226X.2025.42384

**Published:** 2025-02-13

**Authors:** Rutger H. Stoffers, Anne G.H. Niezink, Olga Chouvalova, Jan F. Ubbels, Marleen Woltman-van Iersel, T. Jeroen N. Hiltermann, Lucie B.M. Hijmering-Kappelle, Gea Douma, Sander M. de Hosson, John W.G. van Putten, Friso T. Zandberg, Lisanne V. van Dijk, Johannes A. Langendijk, Robin Wijsman

**Affiliations:** a Department of Radiation Oncology, University Medical Center Groningen, University of Groningen, Groningen, The Netherlands; b Department of Lung Diseases and Tuberculosis, University Medical Center Groningen, University of Groningen, Groningen, The Netherlands; c Treant Hospital, Emmen, The Netherlands; d Wilhelmina Hospital, Assen, The Netherlands; e Martini Hospital, Groningen, The Netherlands; f Ommelander Hospital Group, Scheemda, The Netherlands

**Keywords:** Non-small cell lung cancer, chemoradiotherapy, PD-L1 inhibitors, adverse events, radiation pneumonitis, durvalumab

## Abstract

**Background and purpose:**

Adding adjuvant durvalumab to chemoradiotherapy (CRT) improves overall survival (OS) rates in locally advanced Non-Small-Cell Lung Cancer (NSCLC). However, recent data suggests that this new modality increases the incidence of radiation pneumonitis (RP). The aim of this study was to test the hypothesis that the incidence of RP after CRT and adjuvant durvalumab was higher than after CRT alone among patients with locally advanced NSCLC.

**Materials and methods:**

The study population comprised all patients with NSCLC who completed CRT with curative intent between February 2013 and October 2020. From 2018 on, adjuvant durvalumab was administered in selected patients after completion of CRT. Patient and treatment data together with RP data (CTCAEv4.0, scored up to 9 months after CRT), were prospectively collected as part of our standard follow-up program.

**Results:**

A total of 284 patients were included, of which 90 (30.5%) received adjuvant durvalumab. Incidence of grade ≥2 RP increased in patients receiving durvalumab compared to CRT only (17.8% vs. 8.8%; *p* < 0.05), especially between 6 to 9 months after completing CRT. Adjuvant durvalumab and mean lung dose (MLD) were associated with a higher incidence of grade ≥2 RP (odds ratio [OR]: 2.43 and 1.14, respectively; *p* < 0.05). Current smoking was found to be a protective factor (OR: 0.38; *p* < 0.05).

**Interpretation:**

Adjuvant durvalumab significantly increased the incidence of grade ≥2 RP in this real-world cohort of NSCLC patients. Patients receiving adjuvant durvalumab remain prone to develop grade ≥2 RP longer after completing CRT compared to patients treated with CRT only.

## Introduction

Chemoradiotherapy (CRT) is the standard of care for patients with unresectable stage II to III non-small-cell lung cancer (NSCLC) and good performance status. Concurrent CRT has proven to be superior to sequential administration, improving 5-year overall survival (OS) with 4.5% [1].

The introduction of immune-checkpoint inhibitors (ICI), blocking programmed cell death ligand 1 (PD-L1), has shown to be beneficial in several types of malignancies. The PACIFIC-trial, a multicentre phase III randomized controlled trial, showed that adjuvant durvalumab after concurrent CRT for unresectable stage III NSCLC resulted in a significant improvement of the 5-year OS (from 33% to 43%) and progression free survival (PFS) (from 19.0% to 33.1%) [2, 3].

Toxicity reported in the PACIFIC trial was generally mild, with no significant increase in the incidence of all-cause pneumonitis (including both immune-related pneumonitis [irP] and radiation pneumonitis [RP]): grade ≥2 pneumonitis occurred in 19.4% and 14.1% of the patients treated with adjuvant durvalumab or placebo, respectively [4, 5]. Adjuvant durvalumab treatment was well-tolerated. However, patients participating in randomized controlled trials are generally younger, and often have a better general condition compared to the population in routine clinical practice [6]. Therefore, the question arises if the PACIFIC study outcomes are representative of the outcomes of patients treated with adjuvant durvalumab in clinical practice.

Additionally, retrospective studies have reported on the incidence of treatment-related toxicities, particularly RP, after CRT followed by durvalumab [7–13]. The incidences of RP in these studies, based on real-world data, differed substantially from those reported in the PACIFIC-trial, with grade ≥2 RP incidences varying between 22% and 46% and grade 3/4 RP incidences between 2% and 14%. More studies reporting on toxicity after CRT with and without adjuvant immunotherapy are therefore needed to evaluate RP in ‘real-world’ patients. We compare the incidence of RP and outcomes of locally advanced NSCLC patients treated with CRT with or without adjuvant durvalumab in our study. The aim was to test the hypothesis that RP incidence is increased in patients treated with durvalumab compared to patients treated with CRT only.

## Materials and methods

### Study population and data collection

All patients who completed CRT for NSCLC with curative intent were identified from the Prospective Platform for Evaluation and Development of Lung radiotherapy (ProPED-LUNG; February 2013 to October 2020) [14]. In this database, we prospectively collect data (patient- and treatment characteristics, acute and late toxicity, survival outcomes and quality of life data) of all lung cancer patients treated with curative intent. Data is collected on specific timepoints during and after treatment. Toxicity data, including RP, are reviewed by three dedicated radiation oncologists (not blinded for the treatment received). Toxicity was registered according to pre-defined CTCAE grading in the database. Patients were included if they had histologically confirmed NSCLC treated with fractionated CRT with curative intent. Follow-up time of these patients had to be at least 9 months. Both patients treated with photon or proton beam radiotherapy were included. Patients were excluded in case radiotherapy was given in pre- or postoperative setting and if weekly low-dose gemcitabine was part of the concurrent chemotherapy regimen. For patients treated until 2017, weekly gemcitabine could be part of the concurrent chemotherapy regimen. However, this approach was abandoned as the risk of RP appeared to be higher with this regimen. Because RP is the endpoint of our study, we removed these patients from the evaluation.

### Treatment

Patients underwent radiotherapy in combination with chemotherapy (either concomitant or sequential). Induction chemotherapy was given on discretion of the pulmonologist. Concurrent chemotherapy typically existed as weekly low-dose cisplatin/docetaxel (chemotherapy regimens are listed in Supplementary Material A). The total number of completed chemotherapy cycles was registered.

Radiotherapy consisted of Volumetric Modulated Arc Therapy (VMAT) or Intensity Modulated Radiation Therapy (IMRT). Hybrid forms of these techniques were applied by combining 3-Dimensional conformal radiotherapy (3D-CRT) with VMAT or IMRT. Prescribed doses ranged from 50 to 60 Gy (2.0–2.4 Gy per fraction). From 2019 on, Intensity Modulated Proton Therapy (IMPT) was offered to patients after radiotherapy treatment plan comparison (photon vs. proton radiotherapy) showed to be of benefit in terms of lower toxicity risks concerning the heart, lungs and/or oesophagus [15]. The CRT protocol is described in Supplementary Material A.

From 2018 on, adjuvant durvalumab immunotherapy was administered to patients that received concurrent CRT for unresectable stage III NSCLC (independent of PD-L1 expression) in case of a WHO performance of 0–1 without disease progression after completion of CRT. In some individual cases, durvalumab was given to patients with unresectable stage II and IV (oligometastatic) disease as well. Durvalumab was given in 2-(10 mg/kg) or 4-weekly (20 mg/kg) schedules for up to 1 year or until unacceptable toxicity or disease progression occurred.

### Endpoints and assessments

All patients are followed by their referring pulmonologist according to the Dutch guidelines for lung cancer. The first follow-up moment includes a CT-scan, typically performed within 3 months after CRT in the pre-immunotherapy era and only a few weeks after the end of CRT for those patients who are candidates for adjuvant immunotherapy. Afterwards imaging was performed at least every 6 months in the first 2 years after treatment. All follow-up moments included visits to the out-patient clinic to evaluate treatment-related toxicity. RP was scored according to the CTCAE v4.0, from 6 weeks until 9 months after treatment [16, 17]. RP was scored up to 9 months after ending CRT as we expected that durvalumab treatment might prolong the period of onset of RP. Radiotherapy fractionation data and dose volume histograms (DVH) for organs at risk (OAR) were retrieved from the radiotherapy planning system.

The primary endpoint was the cumulative incidence of grade ≥2 RP (hereafter called RP) in the first 9 months after CRT. The secondary endpoint was 12-month OS from the start of CRT. OS was defined as the time from the first fraction of radiotherapy until death from any cause.

### Statistical analysis

Baseline patient, tumour and treatment characteristics were compared using Mann–Whitney U tests or Chi-square test, depending on their distribution. Logistic regression analysis was performed to analyse the effect of adjuvant durvalumab treatment (and other patients and treatment parameters) on the occurrence of RP. Predictors with a *p*-value <0.2 in univariable regression were used as predictors in the multivariable regression using an enter method. Based on the regression analysis, an NTCP curve was created to illustrate the probability of developing grade ≥2 RP with increasing mean lung dose (MLD) and clinical parameters. The variables with a *p*-value <0.05 in multivariable regression were used in the NTCP curve. The NTCP was calculated based on the equation:


$$NTCP=\frac{1}{1+{\text{e}}^{-(\beta 0+\beta 1*\text{MLD}+\beta 2*\text{var}2+\ldots \beta \text{n}*\text{varn})}}$$

Where, *β*_0_ is the regression coefficient for the intercept (the constant); *β*_1_ is the regression coefficient of the MLD (continuous variable); *β*_2-n_ are the regression coefficients of the binominal variables (var is 0 or 1). In the regression analysis, patients with missing data were excluded from the analysis. OS was analysed using Kaplan–Meier analysis and compared using a log rank test.

Statistical analysis was performed using SPSS v23.0 (Armonk, NY: IBM Corp). A *p*-value <0.05 was considered statistically significant.

## Results

**Figure 1:**
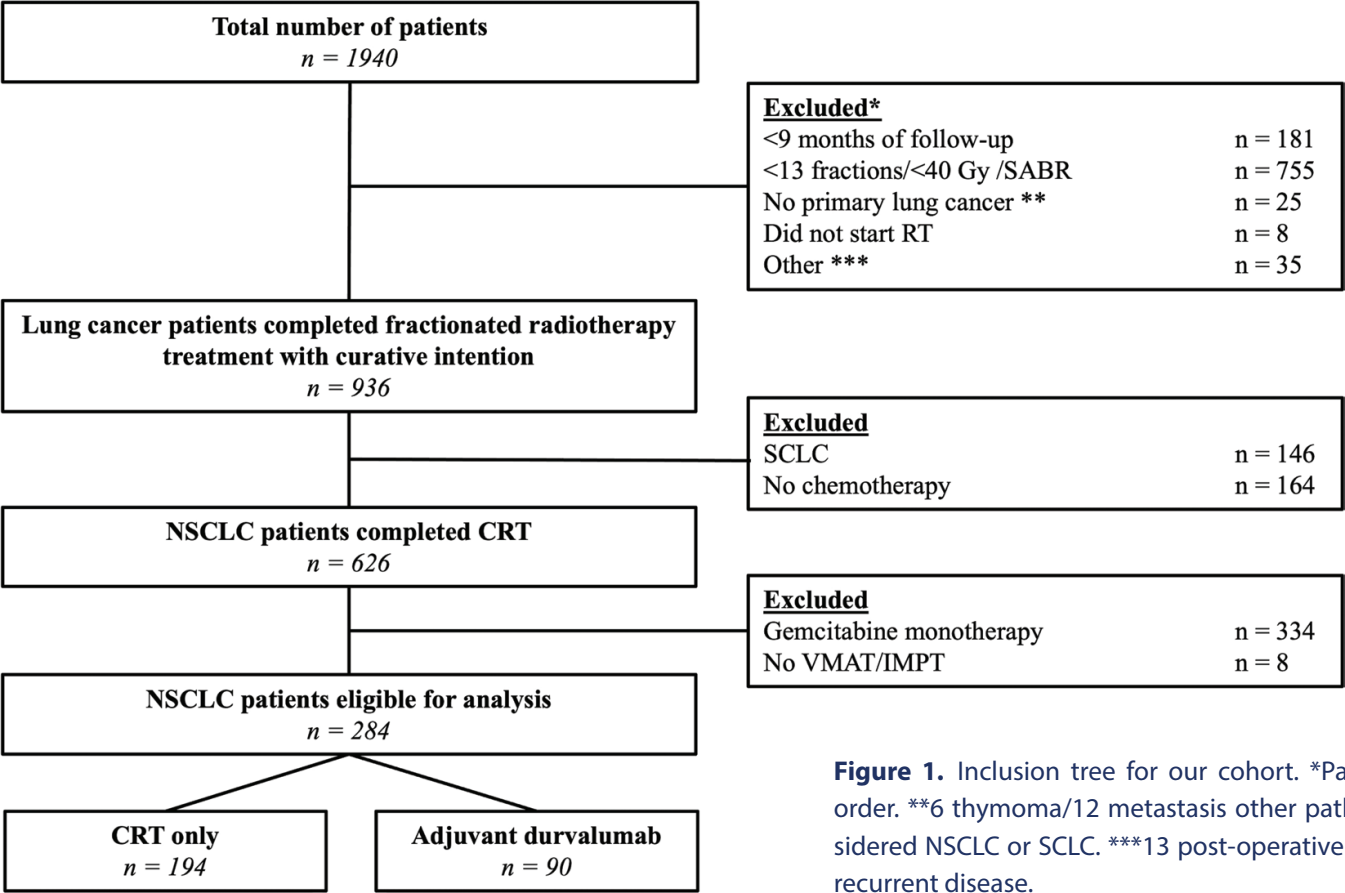
Inclusion tree for our cohort. *Patients were excluded in the listed order. **6 thymoma/12 metastasis other pathology/5 other pathology not considered NSCLC or SCLC. ***13 post-operative RT, 8 trimodality treatment and 13 recurrent disease.

At the time of analysis, 1,940 patients were included in the ProPED-LUNG database. A total of 284 patients met the eligibility criteria, of whom 90 (30.5%) patients received adjuvant durvalumab immunotherapy. Figure 1 shows the flow chart for inclusion of the patients.

Patient and tumour characteristics are listed in Table 1. Baseline patient characteristics did not differ between groups. The median age for the total group was 66.5 years (inter quartile range 60.0–73.0). Most patients receiving adjuvant durvalumab had stage III NSCLC (86.7%).

**Table 1:** Baseline characteristics of the study population

		CRT only (N = 194)	Durvalumab group (N = 90)	p-value
Age	Median (quartiles)	68.0 (60.0–73.0)	65.0 (60.0–71.0)	0.070
	<65 years	77 (39.7)	42 (46.7)	0.166
	65-75	81 (41.8)	39 (43.3)	
	>75 years	36 (18.6)	9 (10.0)	
Gender	Male	117 (60.3)	57 (63.3)	0.626
	Female	77 (39.7)	33 (36.7)	
WHO-PS	0-1	167 (86.1)	77 (85.6)	0.905
	≥2	27 (13.9)	13 (14.4)	
Pulmonary comorbidities	Yes	72 (37.1)	36 (40.0)	0.641
	No	122 (62.9)	54 (60.0)	
Cardiac comorbidities	Yes	88 (45.4)	42 (46.7)	0.837
	No	106 (54.6)	48 (53.3)	
Smoking status	Never	6 (3.1)	2 (2.2)	0.069
	Ex-smoker (<3 months)	22 (11.3)	19 (21.1)	
	Ex-smoker (>3 months)	88 (45.4)	44 (48.9)	
	Current	78 (40.2)	25 (27.8)	
Histology NSCLC	Squamous cell carcinoma	86 (44.3)	38 (42.2)	0.942
	Adenocarcinoma	74 (38.1)	36 (40.0)	
	Other	34 (17.5)	16 (17.8)	
AJCC Stage (8th edition)	I – II	20 (10.3)	8 (8.8)	0.029
	III	145 (74.8)	78 (86.6)	
	IV	29 (14.9)	4 (4.4)	
Chemotherapy sequence	Concurrent	133 (68.4)	85 (94.4)	0.000
	Other	61 (31.4)	5 (5.6)	
Induction chemotherapy type *	Platinum + Gemcitabine	70 (58.8)	39 (53.4)	0.462
	Platinum + Pemetrexed	41 (34.5)	31 (42.5)	
	Other	8 (6.7)	3 (4.1)	
Concurrent chemotherapy type	Platinum + Docetaxel	122 (91.8)	82 (96.5)	0.072
	Other	11 (8.2)	3 (3.5)	
Concurrent chemotherapy complete?	Yes	96 (72.2)	73 (85.9)	0.018
	No	37 (27.8)	12 (14.1)	
RT Technique	Photons	186 (95.9)	62 (68.9)	0.000
	Protons	8 (4.1)	28 (31.1)	
RT Total dose **	<60 Gy	14 (7.2)	1 (1.1)	0.100
	60 Gy	180 (92.8)	89 (98.9)	
Mean Lung Dose	Mean ± SD	11.3 ± 3.7	11. 0 ± 3.3	0.549
Lung V20	<30%	144 (96.6)	71 (98.6)	0.399
	>30%	5 (3.4)	1 (1.4)	
Mean Esophagus Dose	Mean ± SD	16.4 ± 9.5	16.6 ± 8.0	0.602
Mean Heart Dose	Mean ± SD	7.5 ± 6.5	6.7 ± 6.1	0.505

* Patients treated with induction chemotherapy only were categorized as treated with sequential chemotherapy: there are overlapping cases.

** 15 patients received between 50 – 59 Gy: 11 prescribed in 17-25 fraction, 3 patients received <60 Gy by missing one fraction out of 25, 1 patient in the CRT only group was treated with 45 Gy in 30 fractions, twice daily, for a mixed cell carcinoma.

All patients received chemotherapy, either concomitant (with or without induction course) or sequential to radiotherapy. Of all 284 patients, 269 (94.7%) received 60 Gy in 25 once-daily fractions. A total of 36 (12.7%) patients received IMPT. Patients in the adjuvant durvalumab group received significantly more frequent IMPT than patients in the CRT only group (31.1% vs. 4.1%; *p* < 0.01). The MLD was not significantly different between patient treated with photons and patients treated with IMPT (11.4 Gy vs. 9.9 Gy for photons and IMPT, respectively). The MLD and MHD were not significantly different between the durvalumab group and the CRT only group (Table 1).

In total 54 patients (60.0%) received durvalumab in a 2-weekly schedule, of whom 14 (26%) patients completed the full year of adjuvant treatment (median 26 cycles), 14 (26%) patients were still on treatment (median 14 cycles) and in 24 (48%) patients durvalumab treatment was discontinued (median 8 cycles). In total 31 (33%) patients were treated using the 4-weekly regimen of whom 13 patients (42%) completed the full year of treatment (median 15 cycles), 5 (16%) patients were still on treatment (median 11 cycles) and in 13 (42%) patients durvalumab treatment was discontinued (median 8 cycles). For five (5.6%) patients the cycle interval of durvalumab was unknown. Due to the COVID-19 pandemic, some patients (5%) switched from the 2-week schedule to a 4-week schedule.

**Figure 2:**
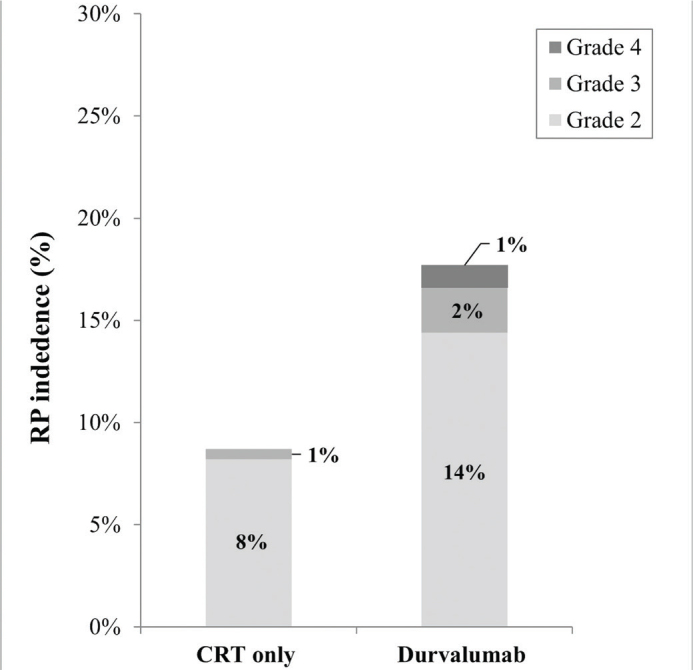
Plot for the incidence of grade ≥ 2 radiation pneumonitis 9 months after ending CRT. The total incidence of grade ≥ 2 radiation pneumonitis was 17.8% in the durvalumab group and 8.8% in the CRT only group. *p < 0.05.

In total 33 patients (11.6%) developed grade ≥2 RP. RP was observed more often in patients receiving adjuvant durvalumab compared to the group receiving CRT only (17.8% vs. 8.8%, *p* = 0.027). Grade 3 RP was observed in two (2%) patients in the durvalumab group and one (0.5%) in the CRT only group. Grade 4 RP was observed in one (1%) patient in the durvalumab group. No grade 5 RP was observed (Figure 2).

**Figure 3:**
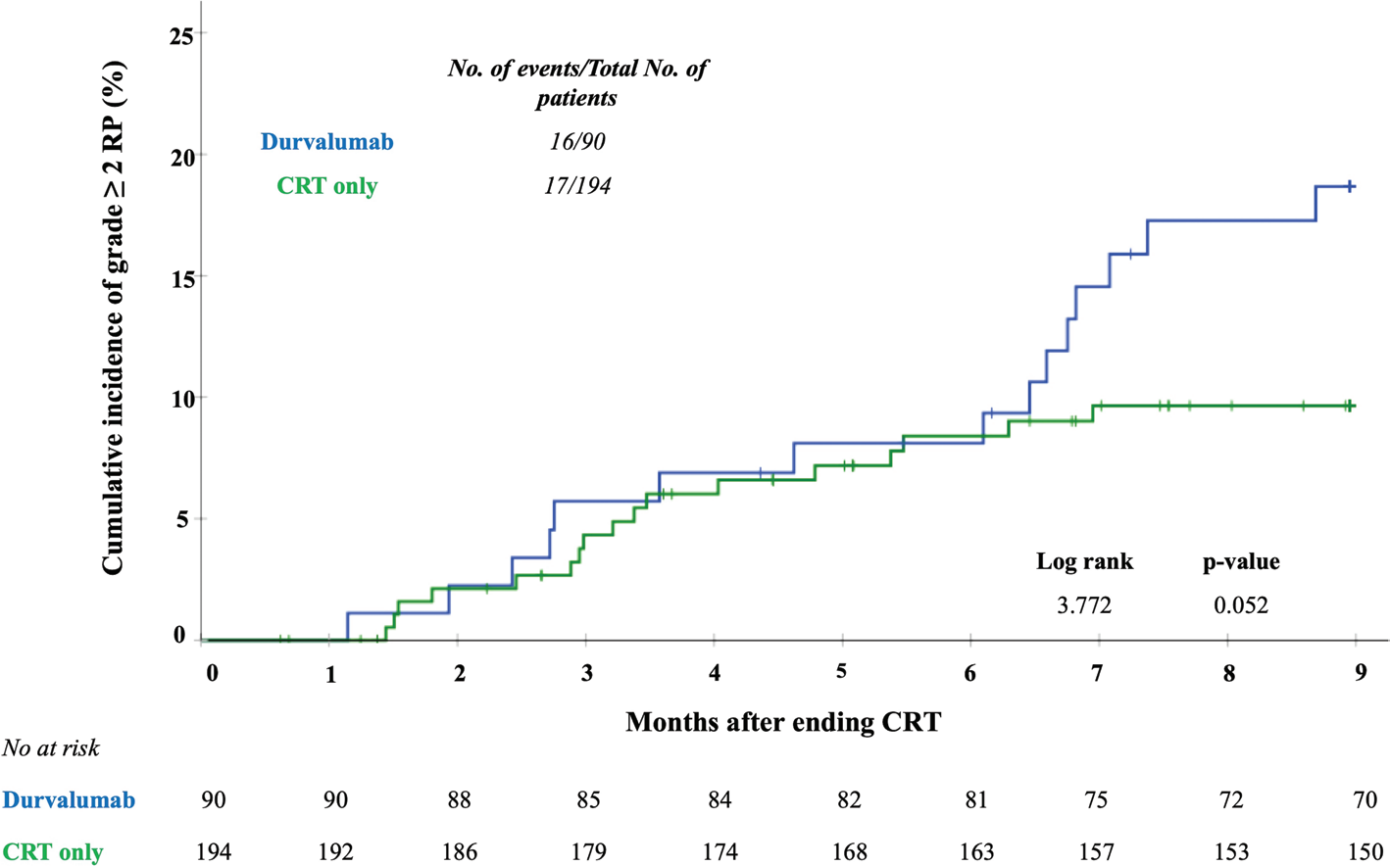
Hazard ratio curves for risk of developing grade ≥2 pneumonitis per week after CRT for adjuvant treatment with durvalumab compared with CRT only. The crossed marks represent the censored data. Analysis was performed at a maximum for 9 months after ending CRT.

Figure 3 shows the cumulative incidence of RP over time. In the first 6 months after CRT the cumulative incidence of RP was similar in both groups. Six months after CRT, no new events of RP were observed in the CRT only group; however, new RP events continued to occur in the durvalumab group. The median time to onset of RP was longer in the durvalumab group compared to the CRT only group: 27.4 (11.9 – 30.6) versus 14.0 (9.3 – 22.1) weeks (*p* = 0.07).

**Figure 4:**
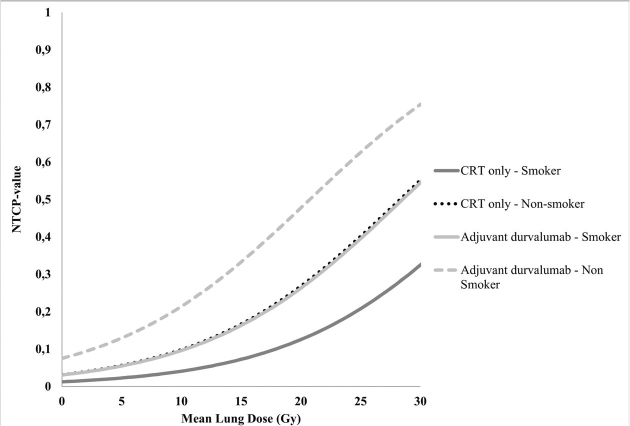
Normal Tissue Complication Probability (NTCP) curves of the probability of developing grade > 2 RP for patients treated with adjuvant durvalumab and smoking status with increasing MLD. [NTCP = 1 / (1 + e^-^ (−4.168 + 0.121*MLD + 0.888*durvalumab treatment + 0.978*smoking status))]. These NTCP values are a representation of our cohort and must be interpreted as such.

In the univariable logistic regression analysis (Table 2), adjuvant durvalumab treatment, the MLD and smoking status were significantly associated with RP (*p* = 0.03, *p* = 0.02 and *p* = 0.04, respectively). In the multivariable logistic regression analysis (Table 2), adjuvant durvalumab (*p* = 0.02) and the MLD (*p* = 0.04) were significantly associated with RP in the first 9 months after radiotherapy with an odds ratio of 2.43 and 1.13 (per Gy), respectively. Current smoking was found to be a protector for RP with an odds ratio of 0.38 (*p* = 0.02). No correlation was observed between adjuvant durvalumab treatment, the MLD and the smoking status. The normal tissue complication probability (NTCP) curves of patients treated with or without durvalumab and their smoking status with increasing MLD are displayed in Figure 4. Treatment technique (VMAT vs. IMPT) was not associated with RP in both univariable and multivariable regression analysis. No correlations were found between IMPT and the predictors for RP.

**Table 2:** Results of univariable and multivariable logistic regression analysis on RP grade ≥ 2 in the first 9 months after ending CRT

			UNIVARIABLE			MULTIVARIABLE		
			OR	95%-CI	p-value	OR	95%-CI	p-value
Age		Continuous	1.011	0.974–1.049	0.562			
		<65 years	1.000	1.000	1.000			
		65-75	0.916	0.421–1.992	0.824			
		>75 years	0.676	0.212–2.159	0.509			
Gender		Male	1.000	1.000	1.000			
		Female	1.005	0.500–2.020	0.989			
WHO		0	1.000	1.000	1.000			
		1	0.913	0.422–1.976	0.818			
		≥2	0.549	0.148–2.040	0.370			
Comorbidities	Pulmonary	Yes	1.000	1.000	1.000			
		No	1.084	0.510–2.303	0.834			
	Cardiac	Yes	1.000	1.000	1.000			
		No	1.298	0.627–2.683	0.482			
	Diabetes	Yes	1.000	1.000	1.000			
		No	0.938	0.340–2.587	0.901			
Smoking status *		Non-smoker	1.000	1.000	1.000	1.000	1.000	1.000
		Smoker	0.380	0.174–0.830	0.015	0.376	0.169–0.837	0.017
Histology		SCC	1.000	1.000	1.000			
		Adenocarcinoma	0.699	0.312–1.566	0.385			
		Other	0.699	0.243–2.011	0.507			
Tumor location	Laterality	Right	1.000	1.000	1.000	1.000	1.000	1.000
		Left	0.490	0.212–1.134	0.096	0.516	0.216–1.233	0.137
		Mediastinal	1.531	0.164–14.289	0.708	1.613	0.153–17.009	0.691
	Lobe	Other	1.000	1.000	1.000			
		Lower	1.292	0.615–2.714	0.499			
Stage		I – II	1.000	1.000	1.000			
		III	0.897	0.290–2.771	0.850			
		IV**	NA	NA	NA			
Chemotherapy	Sequence	Concurrent	1.000	1.000	1.000			
		Other	0.719	0.283–1.826	0.488			
	Induction type	Platinum + Gemcitabine	1.000	1.000	1.000			
		Other	1.220	0.492–3.027	0.667			
	Concurrent type	Platinum + Taxane	1.000	1.000	1.000			
		Other	1.309	0.274–6.252	0.736			
	Complete?	Yes	1.000	1.000	1.000			
		No	0.564	0.185–1.718	0.314			
Radiotherapy	Modality	Photons	1.000	1.000	1.000			
		Protons	1.267	0.456–3.526	0.650			
	DVH	MLD	1.124	1.007–1.255	0.038	1.128	1.005–1.266	0.041
		MHD	0.991	0.937–1.048	0.751			
		MED	0.998	0.958–1.039	0.911			
Immunotherapy	Adjuvant durvalumab	No	1.000	1.000	1.000	1.000	1.000	1.000
		Yes	2.251	1.080–4.693	0.030	2.430	1.124–5.251	0.024

* Smoking status was defined as Smoker when patients were currently smoking or quit smoking <3 months before start of treatment and as Non-smoker if a patient quit smoking >3 months before starting treatment or had no history of smoking.

** No patients with stage IV disease did develop RP.

**Figure 5:**
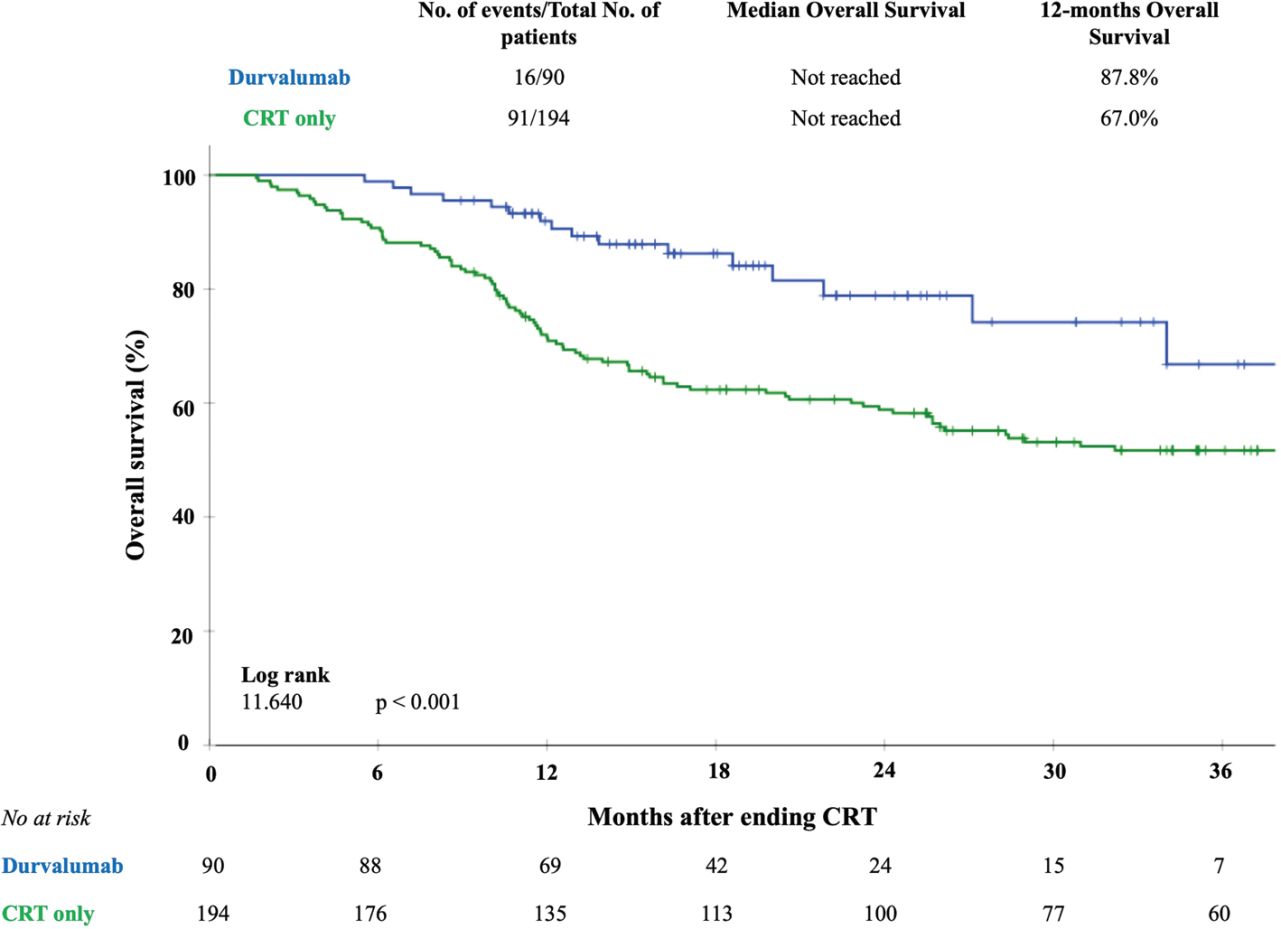
Kaplan–Meier curves of overall survival for adjuvant treatment with durvalumab compared with CRT only. The crossed marks represent the censored data.

The median follow-up of the total number of patients was 25.0 (11.0 – 40.2) months for the CRT only group and 17.5 (12.8 – 25.0) months for the durvalumab group (*p* = 0.08). For the living patients the median follow-up was 39.0 (28.0–49.0) months for the CRT only group and 18.0 (14.0 – 25.0) months for the durvalumab group (*p* = 0.07). From the total of 284 patients, 265 patients (93.3%) had a follow-up time of at least 12 months. The 12-month OS rate was 88% in the durvalumab group, as compared with 67% in the CRT only group (log rank 11.64; *p* < 0.001). Figure 5 shows the OS curves for the total cohort. In a subgroup analysis for OS in patients with stage III disease, the 12-month OS rate was 89% in the durvalumab group, as compared with 68% in the CRT only group (log rank 9.27; *p* < 0.01) (Supplementary Figure 1).

## Discussion

Investigating safety outcomes of new treatment modalities in routine daily practice is important, because clinical trial populations do not always represent ‘real-world’ patients [6]. This analysis showed that in a large prospective longitudinal cohort of NSCLC patients, the incidence of RP increased in patients treated with adjuvant durvalumab, compared to patients treated with CRT only. We found that the time patients are prone to develop RP is prolonged among patients treated with durvalumab compared with patients treated with CRT only, with an increased hazard of RP, especially beyond 6 months after completion of CRT. Moreover, both adjuvant durvalumab and MLD appeared to be independent predictors for RP.

Treatment regimens in our cohort were comparable with the ones used in the PACIFIC trial and all our patients met the radiotherapy dose constraints from that trial (MLD < 20 Gy, lung V20 ≤35% and MED < 34 Gy) [4]. Recently a post-hoc analysis of the PACIFIC trial was published in which a more detailed analysis was provided on the incidence of grade >2 RP and irP and the potential association with baseline factors and clinical outcomes [5]. In this analysis, RP and irP grade >2 was defined as a de novo event that occurred during treatment and within 90 days of the last dose of study medication. The incidence of grade ≥2 pneumonitis in patients receiving durvalumab was 19.8% versus 14.1% in the placebo (CRT only) group. No statistical significance was provided. These rates of pneumonitis are in line with our findings. Median time of onset of pneumonitis between the study groups were similar (70 days vs. 79 days from end of CRT). Interestingly, grade ≥2 pneumonitis was a significant predictor for worse OS, but not for PFS or time to distant metastasis (TTDM). Although this post-hoc analysis provides more insights in pneumonitis development (both RP and irP), no analysis finding predictors using clinical characteristics and dosimetry radiation parameters was performed for the whole trial population. Moreover, as mentioned earlier, the population in clinical trials might not be representative for real world patients [6]. Recently, the first results of the PACIFIC-R study were published, describing an early drug access program for the use of adjuvant durvalumab immunotherapy after CRT in a real-world patient cohort of 1,399 patients with advanced stage NSCLC [18]. A total of 199 patients (14.2%) developed RP for which steroid therapy was indicated. This matches with our finding of 17.8% RP. No comparison to a CRT only cohort was available.

Besides the post hoc PACIFIC analysis, recently, two larger cohort studies were conducted. A large retrospective analysis by Edwards et al. compared the 2-year pneumonitis rates of a historical CRT only cohort (2015–2017; *N* = 989) to a CRT plus durvalumab cohort (2018–2021; *N* = 1005) in stage III NSCLC patients [13]. The incidence of grade >2 pneumonitis (both RP an irP) was 22.1% for the durvalumab group compared to 13.9% in the CRT only group, which was a statistically significant difference. Median time to first onset of pneumonitis was not significantly different between the two groups, although incidence of pneumonitis still increased after 1-year in the durvalumab group and almost stagnated in the CRT only group, which is in line with our study. Durvalumab treatment was found to be a predictor and smoking a protector for developing pneumonitis, which is in line with our study. Unfortunately, dosimetry radiation data analysis was not performed in this study. In a survival analysis, development of grade 3 pneumonitis was associated with worse OS.

Lastly, a retrospective analysis by Yegya-Raman et al. [19] compared a patient cohort of the ICI-era (2017–2021, *N* = 335) to a historic pre-ICI-era cohort (2011–2017, *N* = 448). In the ICI era group, patients were treated with cCRT with or without adjuvant durvalumab. The 1-year RP rate was 31.4% for patients treated in the ICI-era compared to 20.1% in the historic cohort. Interestingly, within the ICI-era group, patients treated with adjuvant durvalumab were more prone to develop RP, with a longer median time to occurrence of RP compared to patients treated with cCRT only (5.0 vs. 3.6 months respectively), which is in line with our findings. They also found a significant association between RP and MLD and lung V20, similar to our results. No clinical factors were associated with increasing RP.

To our knowledge, our study is the first to provide an analysis in which clinical parameters, chemotherapy data, dosimetric radiotherapy data and immunotherapy treatment data were analysed all together to find predictors for grade ≥2 RP. In multivariable analysis, MLD, smoking status and adjuvant durvalumab were significantly associated with RP. The MLD is a well-known predictor for RP after radiotherapy for intra-thoracic tumours [20, 21]. A meta-analysis by Vogelius et al. showed that clinical factors such as older age, presence of (pulmonary) comorbidities and the primary tumour located in the inferior or middle part of the lung were risk factors for developing RP [22]. In our cohort, we did not find these relationships. However, Vogelius et al. also reported that current tabaco smoking was protective for RP, which is in line with our findings. The exact mechanisms for this are not clear. A previous study reported a diminished radiation-induced inflammatory response in the lung in active smokers irradiated for breast cancer [23]. This smoking induced intrapulmonary immunosuppression might be a possible explanation for the fact that active smokers have a reduced sensitivity of the lung to radiation and RP. Moreover, a more hypoxic environment in smokers might lead to reduced radiation-induced lung damage, because of less generation of reactive oxygen species leading to DNA damage [24]. It must be noted that the interpretation of this result requires careful consideration, since the analysis did not account for severity of pulmonary comorbidities or pulmonary function. For NSCLC patients, prediction models have been built based on the previously mentioned clinical parameters and dosimetry data [25, 26]. However, no studies have reported durvalumab treatment as a predictor in a model for RP yet. Based on our findings, adjuvant immunotherapy should be considered as a predictive factor for RP in future RP risk modelling.

With IMPT, the mean dose to OARs such as the lungs can be significantly reduced, which in turn may decrease the risk of RP [27, 28]. Most patients treated with IMPT (78%) in our cohort were in the durvalumab group. Intensity Modulated Proton Therapy was not associated with RP. However, the small number of patients receiving IMPT (12.7%) hampers sound evaluation of the effect of IMPT on the incidence of RP.

Adjuvant durvalumab significantly improved 12-months OS compared to CRT only: 88% versus 68%. The 12-months OS in the durvalumab group is in line with the OS reported 84.3% in the PACIFIC trial. Although the design of our study makes comparing survival data of our cohort to the PACIFIC trial a challenge, adjuvant durvalumab treatment is very likely to be beneficial for real-world NSCLC patients, as shown by the PACIFIC-R trail. In contrast, previously mentioned studies described an association between developing high grade pneumonitis and worse OS [5, 13]. This emphasises the need for better understanding of the development of both RP and irP to limit this toxicity as much as possible.

A potential study limitation is the patient selection bias and imbalances in the predictive factors between the cohorts. For example, patients selected for adjuvant durvalumab treatment tended to be younger than patients treated with CRT only (median age 64 vs. 68 years *p* = 0.07). Patients in the CRT only group were treated with sequential chemotherapy more often. Both factors may influence the RP incidences. Furthermore, patients in the durvalumab group were more therapy compliant and completed concurrent chemotherapy more often (86% vs. 72% *p* = 0.02), which is possibly related to patient performance.

Another limitation may be that PD-L1 inhibitors in themselves can cause pneumonitis in NSCLC patients [29]. Distinguishing RP from irP in patients treated with both modalities is very challenging and development of symptomatic RP and irP often leads to (temporary) discontinuation of adjuvant ICI-treatment. Specific changes in lung tissue as seen on follow-up imaging have been described for both RP and irP [30–32]. Probably, our reported pneumonitis rates are a mix of RP, irP or the combination of both, which may partly explain why the cumulative RP incidence curves separate at 6 months after treatment (Figure 3). In our database, nine patients developed RP between 6 and 9 months after ending CRT. Six of them developed RP solely within the high and intermediate radiation dose areas in the lung (tumour dose – V20 of the lung). The other three developed pneumonitis within the radiotherapy area, but also in areas of the lung not treated by radiotherapy (different lobes or the untreated contralateral lung). No consistent pattern could be discovered. Further research, using radiological features (radiomics) and possibly artificial intelligence, might help to adequately distinguish the different types of pneumonitis in this patient group.

In conclusion, we found an increased incidence of grade ≥2 RP in NSCLC patients receiving adjuvant durvalumab immunotherapy. The onset of RP continued 6 months after the end of radiotherapy in this group of patients, in contrast to the patients treated with CRT only. Durvalumab treatment showed, besides known clinical and radiotherapy dosimetry factors, to be a predictive factor for developing RP. Therefore, adjuvant immunotherapy should be considered as a predictive factor in future modelling of the risk of RP.

## Supplementary Material

Rising incidence of radiation pneumonitis after adjuvant durvalumab in NSCLC patients treated with concurrent chemoradiotherapy

## Research availability statement

Research data is available upon request.
