# Supplementary material for: Rising incidence of radiation pneumonitis after adjuvant durvalumab in NSCLC patients treated with concurrent chemoradiotherapy
Source: Acta Oncol. 2025 Feb 13;64:42384. doi: 10.2340/1651-226X.2025.42384 (PMC11848943; doi:10.2340/1651-226X.2025.42384)
Supplement: Rising incidence of radiation pneumonitis after adjuvant durvalumab in NSCLC patients treated with concurrent chemoradiotherapy [file AO-64-42384-s1.pdf]

Supplementary material has been published as submitted. It has not been copyedited, or typeset by Acta Oncologica

Supplement A

Radiotherapy protocol

Before start of treatment, adequate tumor staging was performed according to the Dutch guideline for lung cancer. A FDG-PET-scan and/or CT-scan of the thorax, along with lymph node staging using bronchoscopy, EUS/EBUS and/or mediastinoscopy was performed.

Radiotherapy Planning CT-scan

A planning 4D CT scan is performed using breathing registration and contrast agent on discretion of the radiation oncologist. A 10-phase reconstruction is made, along with an Maximum-intensity-projection (MIP) and an average projection. The average projection is used for target delineation and is matched with the diagnostic FDG-PET-scan or CT-scan of the thorax.

Target delineation

- Gross tumor volume (GTV) consist of the primary tumor (GTVtumor) and the pathologic lymph nodes (GTVnode) found in the diagnostic work-up. The GTV is delineated based on the volumes in expiration.
- The internal gross tumor volume (IGTV) consist of the GTV, expanded based on the 10 different phases of the 4D-CT scan (or the MIP reconstruction) to account for the tumor movement during the breathing cycles.
- The clinical target volume (CTV) accounts for the microscopic extension around the tumor and consists of the IGTV with a margin of 5 mm. This extension is, when necessary, adapted to anatomical boundaries (i.e. thoracal wall, trachea or vertebrae).
- (Photons) The planning target volume (PTV) accounts for the uncertainty for patient movement between radiation fractions and consist of the CTV with a 6mm margin.

Organs at risk (OAR) delineation

- Myelum: the total spinal canal
- Lungs: both lungs are delineated separately. The total lung volume is bases on the summed volume of both lungs minus the GTV
- Heart: the total heart is delineated according to Feng et al.
- Esophagus: the esophagus is delineated from the cricoid to the gastro-esophageal junction
- Brachial plexus: neuroforamen C4-C5 to Th1-Th2. Medially from the vertebrae to laterally to the medial scalene muscle. The caudal border is the subclavian vein.

Clinical goals

Photon therapy (VMAT of partial VMAT (hybrid forms of VMAT and IMRT/3D-CRT)

Dose to the PTV: D98 = 95% of the prescribed dose. On individual cases this goal can be adjusted.

Proton therapy (IMPT)

Proton therapy is planned robust using a 24-scenario planning.

Evaluation of the target area (CTV):

- Nominal plan: D50 and the mean prescription dose ± 1 Gy (aim is 0.5 Gy)
- Voxel wise min: CTV V95 ≥ 98%

Dose constraints

- Myelum: maximum dose (D1) = 50 Gy (25 fraction) (a/b = 2)
- Lungs: the constraint dose on the lungs is bases on the total lung min the GTV volume
    - Mean Lung Dose (MLD) ≤ 20 Gy
    - V20 ≤ 30%
    - V5 ≤ 70%
- Esophagus: as low as reasonably possible (ALARA); V35 ≤ 65%
- Heart
    - Mean Heart Dose ≤ 26 Gy
    - V50 ≤ 25%
    - V30 ≤30%
- Brachial plexus: maximum dose (D1) = 60 Gy (25 fractions)

Chemotherapy protocol

Induction chemotherapy

Induction chemotherapy was given on discretion of the pulmonologist and based on patient characteristics (i.e.performance score). It generally consisted of platinum based chemotherapy (either cisplatin of carboplatin) combined with either pemetrex or gemcitabine based on the histological subtype. Mostly two 2-weekly cycles were administered.

- Squamous cell carcinoma: cisplatin 80mg/m^2 or carboplatin AUC 6 + gemcitabine 1125 mg/m^2
- Adenocarcinoma: cisplatin 80mg/m^2 or carboplatin AUC 6 + pemetrexed 500mg/m^2
- For other histological subtypes (other than small-cell lung cancer) or when deviating from the protocol: cisplatin 80mg/m^2 or carboplatin AUC 6 + etoposide

Concurrent chemoradiotherapy

Chemoradiotherapy was preferable given concurrent based on the patients performance score as a radiosensitizer. It generally consisted on platinum based chemotherapy (either cisplatin or carboplatin) combined with a taxane.

- Weekly: cisplatin 20mg/m^2 or carboplatin + Docetaxel 20mg ^{2}

Sequential chemoradiotherapy

Sequential chemoradiotherapy was administered when concurrent chemoradiotherapy was found to toxic based on patient characteristics (i.e. age and performance score) and in individual cases on tumor size and location. It typically consists on platinum based chemotherapy combined with either pemetrex or gemcitabine based on the histological subtype. Cycles were given weekly.

- Squamous cell carcinoma: cisplatin 80mg/m^2 or carboplatin AUC 6 + gemcitabine 1125 mg/m^2
- Adenocarcinoma: cisplatin 80mg/m^2 or carboplatin AUC 6 + pemetrexed 500mg/m^2
- For other histological subtypes (other than small-cell lung cancer) or when deviating from the protocol: cisplatin 80mg/m^2 or carboplatin AUC 6 + etoposide

Supplementary Figure 1

	No. of events/Total No. of patients	Median Overall Survival	12-months Overall Survival
**Durvalumab**	9/78	Not reached	88.5%
**CRT only**	46/145	Not reached	68.3%

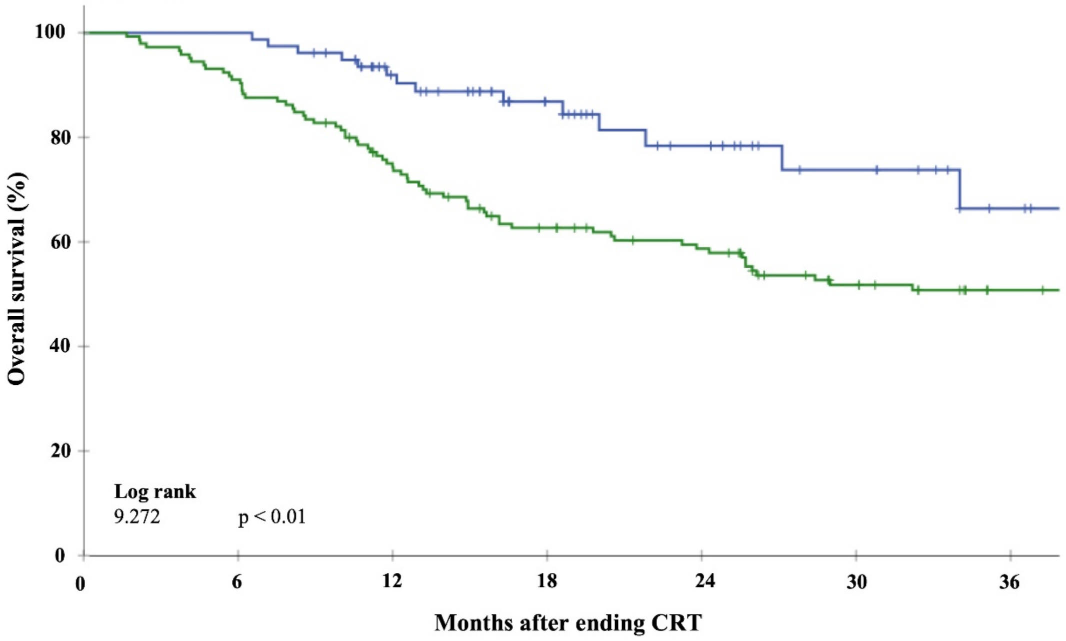

No at risk							
**Durvalumab**	78	78	58	36	24	15	7
**CRT only**	145	132	104	83	73	55	44

Supplementary figure 1 subgroup survival analysis for stage III patients. Kaplan-Meier curves of overall survival for adjuvant treatment with durvalumab compared with CRT only. The crossed marks represent the censored data.
